# Gut microbiota dysbiosis contributes to the development of hypertension

**DOI:** 10.1186/s40168-016-0222-x

**Published:** 2017-02-01

**Authors:** Jing Li, Fangqing Zhao, Yidan Wang, Junru Chen, Jie Tao, Gang Tian, Shouling Wu, Wenbin Liu, Qinghua Cui, Bin Geng, Weili Zhang, Ryan Weldon, Kelda Auguste, Lei Yang, Xiaoyan Liu, Li Chen, Xinchun Yang, Baoli Zhu, Jun Cai

**Affiliations:** 1 Hypertension Center, Fuwai Hospital, State Key Laboratory of Cardiovascular Disease of China, National Center for Cardiovascular Diseases of China, Chinese Academy of Medical Sciences and Peking Union Medical College, Beijing, 100037 China; 2 Department of Cardiology, Beijing ChaoYang Hospital, Capital Medical University, Beijing, 100020 China; 3 Beijing Key Laboratory of Hypertension, Beijing, 100020 China; 4 Computational Genomics Laboratory, Beijing Institutes of Life Science, Chinese Academy of Sciences, Beijing, 100101 China; 5 Novogene Bioinformatics Institute, Beijing, 100000 China; 6 Department of Cardiology, Baoding NO.1 Central Hospital, Baoding, 071000 China; 7 Department of Cardiology, The First Affiliated Hospital, Xi’an Jiaotong University, Xi’an, 710061 China; 8 Department of Cardiology Kailuan General Hospital, Hebei Union University, Tangshan, 063000 China; 9 Department of Biomedical Informatics, Centre for Noncoding RNA Medicine, School of Basic Medical Sciences, Peking University, Beijing, 100191 China; 10 Department of Biology and Biochemistry, University of Houston, Houston, TX 77204 USA; 11 Medical Research Center, Beijing ChaoYang Hospital, Capital Medical University, Beijing, 100020 China; 12 Department of Stem Cell Engineering, Texas Heart Institute, Houston, TX 77030 USA; 13 Tongji Hospital, Huazhong University of Science and Technology, Wuhan, Hubei 430030 China; 14 CAS Key Laboratory of Pathogenic Microbiology and Immunology, Institute of Microbiology, Chinese Academy of Sciences, Beijing, 100101 China; 15 Collaborative Innovation Center for Diagnosis and Treatment of Infectious Diseases, The First Affiliated Hospital, College of Medicine, Zhejiang University, Hangzhou, 310003 China

**Keywords:** Hypertension, Pre-hypertension, Gut microbiota, Metabolism, Fecal transplant

## Abstract

**Background:**

Recently, the potential role of gut microbiome in metabolic diseases has been revealed, especially in cardiovascular diseases. Hypertension is one of the most prevalent cardiovascular diseases worldwide, yet whether gut microbiota dysbiosis participates in the development of hypertension remains largely unknown. To investigate this issue, we carried out comprehensive metagenomic and metabolomic analyses in a cohort of 41 healthy controls, 56 subjects with pre-hypertension, 99 individuals with primary hypertension, and performed fecal microbiota transplantation from patients to germ-free mice.

**Results:**

Compared to the healthy controls, we found dramatically decreased microbial richness and diversity, *Prevotella*-dominated gut enterotype, distinct metagenomic composition with reduced bacteria associated with healthy status and overgrowth of bacteria such as *Prevotella* and *Klebsiella*, and disease-linked microbial function in both pre-hypertensive and hypertensive populations. Unexpectedly, the microbiome characteristic in pre-hypertension group was quite similar to that in hypertension. The metabolism changes of host with pre-hypertension or hypertension were identified to be closely linked to gut microbiome dysbiosis. And a disease classifier based on microbiota and metabolites was constructed to discriminate pre-hypertensive and hypertensive individuals from controls accurately. Furthermore, by fecal transplantation from hypertensive human donors to germ-free mice, elevated blood pressure was observed to be transferrable through microbiota, and the direct influence of gut microbiota on blood pressure of the host was demonstrated.

**Conclusions:**

Overall, our results describe a novel causal role of aberrant gut microbiota in contributing to the pathogenesis of hypertension. And the significance of early intervention for pre-hypertension was emphasized.

**Electronic supplementary material:**

The online version of this article (doi:10.1186/s40168-016-0222-x) contains supplementary material, which is available to authorized users.

## Background

In recent decades, the potential role of the gut microbiome in altering health status of the hosts has drawn considerable attention. Emerging evidence suggests a link between gut microbiome and various diseases, including colorectal cancer, liver cirrhosis, arthritis, type 2 diabetes, and atherosclerosis [1–5]. A number of microbial biomarkers specific to these diseases have been discovered, and fecal microbiome-targeted strategies are recommended to be a powerful tool for early diagnosis and treatment of different diseases.

More importantly, by fecal transfer experiment and gut microbiota (GM) remodeling, intestinal microbiome has been further indicated to conduce to the pathogenesis of multiple diseases such as obesity, depressive disorder, chronic ileal inflammation, liver diseases, and atherosclerosis [6–12]. Specific mechanisms underlying the causal function of GM have been revealed. For example, the metabolism by intestinal microbiota of dietary L-carnitine, a nutrient in red meat, was demonstrated to promote atherosclerosis and lead to cardiovascular disease risk via producing trimethylamine and trimethylamine-N-oxide [12]. Targeting gut microbial production of trimethylamine specifically and non-lethal microbial inhibitors were confirmed to relieve diet-induced atherosclerotic lesion development [13]. Thus GM may serve as a potential therapeutic approach for the treatment of cardiovascular and metabolic diseases.

Hypertension (HTN) has become a global public health concern and a major risk factor for cardiovascular, cerebrovascular, and kidney diseases [14, 15]. It is believed that the etiology of HTN depends on the complex interplay of both genetic and environmental factors [16, 17], and the precise cause of this morbidity has not been elucidated to date. It has been suggested that the germ-free (GF) mice, in which the intestinal bacteria is completely absent, present relatively lower blood pressure (BP) when compared to conventional mice [18]. And therefore we suspected that GM might have the potential to regulate BP.

Most recently, many lines of seminal evidence, which for the first time demonstrate that aberrant gut microbial community are linked to BP changes of the host, support this hypothesis. For example, disordered GM as a result of decreased microbial richness, diversity, evenness, and increased *Firmicutes*/*Bacteroidetes* ratio was reported in hypertensive animals and seven HTN patients, as sequenced by 16S ribosomal RNA [19]. In Dahl rats, distinct metagenomic composition have been revealed between salt-sensitive and salt-resistant strains, and the GM of salt-sensitive rats was suggested to be in a symbiotic relationship with the host [20]. In addition, by rat models of HTN and meta-analyses in randomized human clinical trials, investigators have revealed that administration of probiotics can reduce BP [21, 22]. This drove us to speculate that the alteration in GM by probiotic use may lead to BP changes. Furthermore, it has been proved that transplantation of cecal contents from hypertensive obstructive sleep apnea rats on high-fat diet into recipient rats on normal chow diet lead to higher BP levels, and a major contributor to the gut dysbiosis of obstructive sleep apnea-induced HTN is high-fat diet [23]. These studies have emphasized a strong correlation between gut dysbiosis and HTN, and further implied the significance of GM in BP regulation, yet animal models could not perfectly substitute human disease, and the sample size of human participants for microbial analysis was quite limited.

In consideration of the BP levels being classified into optimal, pre-hypertension (pHTN), and HTN according to the most recent clinical guidelines [24], it remains obscure how exactly the composition of gut microbes and the products of microbial fermentation change in human patients with HTN, especially in pHTN populations. In addition, decisive evidence is still needed to determine whether gut dysbiosis is a consequence or an important causal factor for the pathogenesis of HTN. Fecal transplantation from human samples into GF mice is required to uncover the involvement of GM dysbiosis in pathophysiology of HTN. Collectively, these key issues are the major goal of the present study.

To address the questions above, we performed deep metagenomic sequencing of stool samples from 196 participants of healthy control, pHTN, and HTN; took metabolomic analyses of their metabolic profiles, further constructed a disease classifier for pHTN and HTN based on GM and metabolites; and demonstrated the crucial role of disordered GM in triggering thigh BP by human fecal microbiota transplantation into GF mice.

## Results

### GM diversity and enterotype in pHTN and HTN

To identify whether gut microbial changes are associated with HTN, we performed shotgun metagenomic sequencing of fecal samples from a cohort of 196 Chinese individuals. The cohort consisted of 41 healthy controls, 56 subjects with pHTN, and 99 patients with primary HTN. All the participants were from a cohort study among employees of the Kailuan Group Corporation. The Kailuan study is a prospective cohort study focusing on the Kailuan community in Tangshan, a large modern city in northern China. All the subjects in the hypertension group were newly diagnosed hypertensive patients prior to antihypertensive treatment. Patients suffering from cancer, heart failure, renal failure, smoking, stroke, peripheral artery disease, and chronic inflammatory disease were all excluded. Drugs including statins, aspirin, insulin, metformin, nifedipine, and metoprolol were not used on the patients, and other drug consumption was not compared because the sample size was quite small. Individuals were also excluded if they had received antibiotics or probiotics within the last 8 weeks. Other than SBP and DBP, there was no significant difference in other clinical parameters among groups, except for fasting blood glucose level (FBG) (*P* = 0.026, C vs H; Kruskal-Wallis test, Additional file 1: Table S1). Bacterial DNA was extracted from stool samples, sequenced on the Illumina platform, and a total of 1211 Gb 125-bp paired-end reads were generated, with an average of 6.18 ± 1.43 (s.d.) million reads per sample (Additional file 2: Table S2). For each sample, a majority of high-quality sequencing reads (83.74–97.24%) were de novo assembled into long contigs or scaffolds, which were used for gene prediction, taxonomic classification, and functional annotation.

**Fig. 1:**
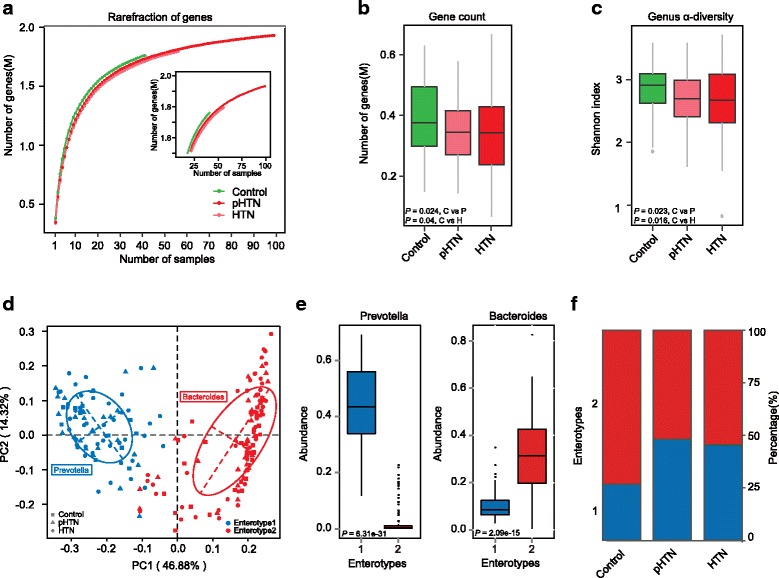
Decreased diversity and shift of gut enterotypes in human adults with pHTN and HTN. **a** Rarefaction curves for gene number in control (*n* = 41), pHTN (*n* = 56), and HTN (*n* = 99) after 100 random sampling. The curve in each group is near smooth when the sequencing data are great enough with few new genes undetected. **b, c** Comparison of the microbial gene count and *α* diversity (as accessed by Shannon index) based on the genera profile in the three groups. C, control; P, pHTN; H, HTN. *P* = 0.024, C vs P; *P* = 0.04, C vs H; for gene count. *P* = 0.023, C vs P; *P* = 0.016, C vs H; for *α* diversity. *P* values are from Kruskal-Wallis test. **d** A total of 196 samples are clustered into enterotype 1 (*blue*) and enterotype 2 (*red*) by PCA of Jensen-Shannon divergence values at the genus level. The major contributor in the two enterotypes is *Prevotella* and *Bacteroides*, respectively. **e** Relative abundances of the top genera (*Prevotella* and *Bacteroides*) in each enterotype. *P* = 6.31e−31 and *P* = 2.09e−15, respectively; Wilcoxon rank sum test. **f** The percentage of control, pHTN and HTN samples distributed in two enterotypes. 26.83% normotensive controls, 48.21% pHTN, and 45.45% HTN are found in enterotype 1. *P* = 0.02, C vs P; *P* = 0.03, C vs H; Fisher’s exact test. *Boxes* represent the inter quartile ranges, the *inside line or points* represent the median, and *circles* are outliers

To characterize the bacterial richness, rarefaction analysis was performed by randomly sampling 100 times with replacement and estimating the total number of genes that could be identified from these samples. The curve in each group was near saturation, which suggested the sequencing data were great enough with very few new genes undetected. The rate of acquisition of new genes in control samples rapidly outpaced new gene acquisition in disease samples, suggesting lower levels of gene richness in the pHTN and HTN groups (Fig. 1a). The number of genes in both pHTN and HTN groups were significantly decreased as compared to the controls (*P* = 0.024, C vs P; *P* = 0.04, C vs H; Kruskal-Wallis test, Fig. 1b). Shannon index based on the genera profile was calculated to estimate the within-sample (*α*) diversity. Consistently, the *α* diversity at the genus level was much lower in pHTN and HTN groups (*P* = 0.023, C vs P; *P* = 0.016, C vs H; Kruskal-Wallis test, Fig. 1c). The reduced richness of genes and genera in the GM of pHTN and HTN groups is consistent with previous findings [19], suggesting possible deficiency of healthy microflora in hypertensive patients.

To explore the difference between the microbial communities at different stages of HTN, enterotypes were identified based on the abundance of genera using Partitioning Around Medoid (PAM) clustering method. The optimal number of enterotypes was two as indicated by Calinski-Harabasz (CH) index (Additional file 3: Figure S1). Then Principal Coordinate Analysis (PCoA) using Jensen-Shannon distance was performed to cluster the 196 samples into two distinct enterotypes (Fig. 1d). *Prevotella* was the most enriched genus in enterotype 1; *Bacteroides* was the most enriched genus in enterotype 2 (*P* = 6.31e−31 and *P* = 2.09e−15, respectively; Wilcoxon rank sum test, Fig. 1e). Both contributors in the two enterotypes have been reported in European and Chinese populations before [2, 3]. There was a higher percentage of pre-hypertensive and hypertensive patients distributed in enterotype 1 (48.21% for pHTN, and 45.45% for HTN), while more healthy controls (73.17%) were found in enterotype 2 (*P* = 0.02, C vs P; *P* = 0.03, C vs H; Fisher’s exact test; Fig. 1f). These findings suggest that enterotype 2 may represent a GM community structure associated with healthy control, while enterotype 1 may be associated with pHTN and HTN.

Considering the higher percentage of HTN patients in enterotype 1, we clustered the genera in this enterotype and further explored the microbial co-occurrence network by Spearman’s correlation. There was a positively interacted network constituted by 12 genera, which were negatively correlated with *Prevotella*, the core genus in this enterotype (Additional file 4: Figure S2a). All these genera were decreased in enterotype 1 as compared with enterotype 2 (Additional file 4: Figure S2b). Eight out of them were directly linked to *Prevotella*, while the other four, including *Oscillibacter*, *Faecalibacterium*, *Butyrivibrio*, and *Roseburia*, were indirectly linked to *Prevotella*. These findings highlighted the possibility of *Prevotella* as a key genus associated with pHTN and HTN. The difference in gut enterotype distribution revealed profound changes of the intestinal microbiome structure in both pHTN and HTN, implying the significance of gut microbes in the development of HTN.

### pHTN and HTN-associated genera in GM

**Fig. 2:**
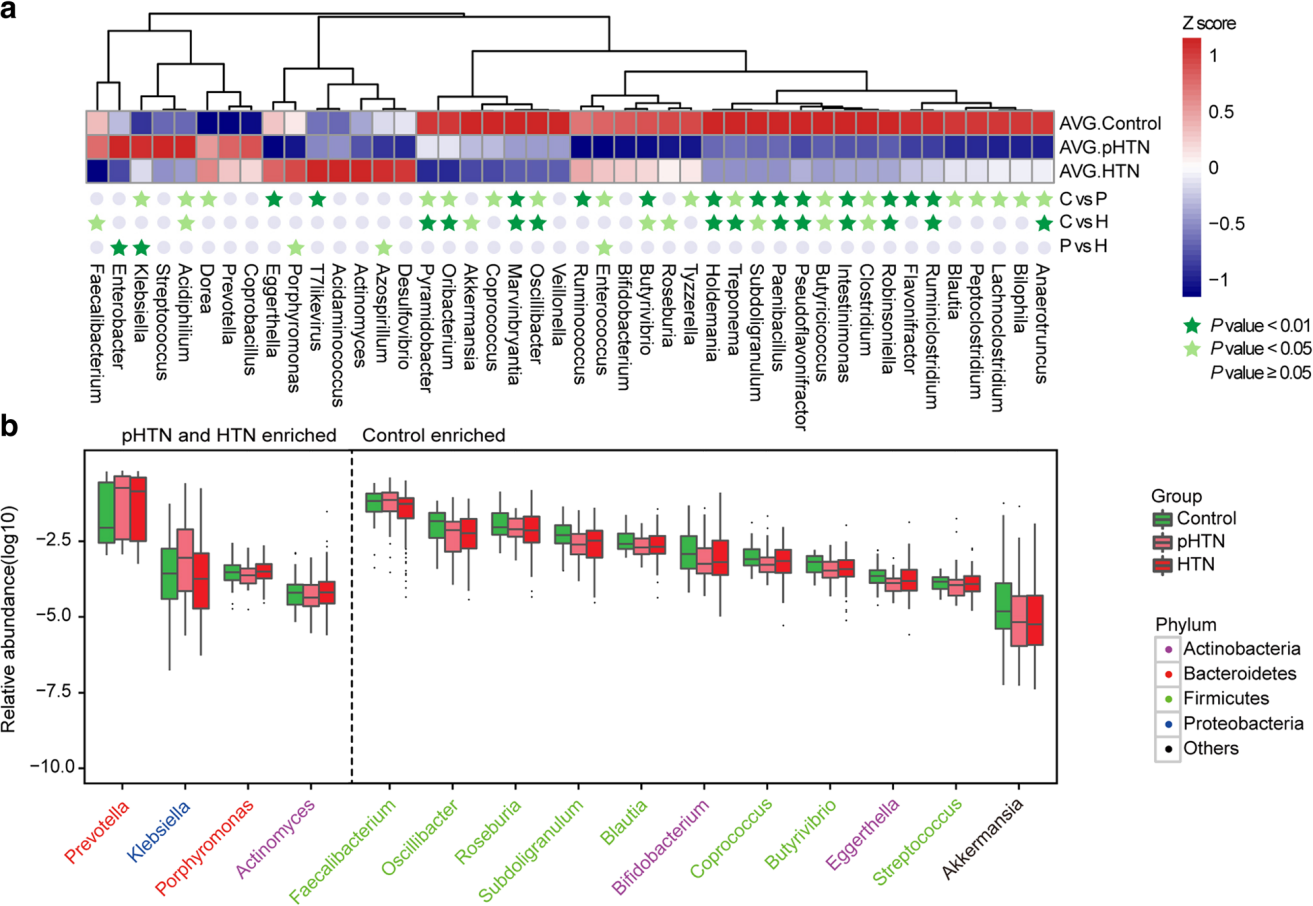
Genera strikingly different across groups. **a** Relative abundance of the top 44 most different genera across groups at the criteria of *P* value <0.1 by Wilcoxon rank sum test. C, control; P, pHTN; H, HTN. The abundance profiles are transformed into Z scores by subtracting the average abundance and dividing the standard deviation of all samples. Z score is negative (shown in *blue*) when the row abundance is lower than the mean. Genera at *P* value <0.01 are marked with *dark green star*, *P* value <0.05 with *light green star*, and *P* value ≥0.05 with *gray circle*. **b** The *box plot* shows the relative abundance of four genera enriched in pHTN and HTN patients, and 11 genera abundant in control. Genera are colored according to the phylum. *Boxes* represent the inter quartile ranges, *lines inside the boxes* denote medians, and *circles* are outliers

Genes were aligned to the NR database and annotated to taxonomic groups. The relative abundance of gut microbes was calculated by summing the abundance of genes as listed in Additional file 2: Table S3–S4. *P* values were tested by Wilcoxon rank sum test and corrected for multiple testing with Benjamin & Hochberg method as others previously did [4, 25]. It is worth mentioning that 44 genera were differentially enriched in control, pHTN, and HTN (*P* < 0.1, Wilcoxon rank sum test, Fig. 2a and Additional file 2: Table S5). Fifteen of them were further shown in Fig. 2b. Genera such as *Prevotella* and *Klebsiella* were overrepresented in individuals with pHTN or HTN (Fig. 2b). *Prevotella*, originated from mouth and vagina, was abundant in the microbiome of our study cohort. The pathogenesis of periodontal diseases and rheumatoid arthritis are thought to be attributed to *Prevotella* [3, 26]. A wide range of infectious diseases are known to be attributed to *Klebsiella* [27, 28]. *Porphyromonas* and *Actinomyces*, which were also elevated in the HTN group, are morbific oral bacteria that cause infections and periodontal diseases [29].

By contrast, *Faecalibacterium*, *Oscillibacter*, *Roseburia*, *Bifidobacterium*, *Coprococcus*, and *Butyrivibrio*, which were enriched in healthy controls, declined in pHTN and HTN patients (Fig. 2b). Our observations were consistent with the genera negatively correlated with *Prevotella* in the network of enterotype 1 (Additional file 4: Figure S2), and these bacteria are known to be essential for healthy status. For example, reduced levels of *Faecalibacterium* and *Roseburia* in the intestines are associated with Crohn’s disease and ulcerative colitis [30, 31]. Both bacteria are crucial for butyric acid production [30, 32]. Moreover, *Bifidobacterium* is an important probiotic necessary to intestinal microbial homeostasis, gut barrier, and lipopolysaccharide (LPS) reduction [33].

The divergence of GM composition in each sample was assessed to explore the correlation of microbial abundance with body mass index (BMI), age, and gender (Additional file 5: Figure S3). Although the gender ratio is discrepant among groups (Additional file 1: Table S1), we found no remarkable regularity of bacterial abundance based on BMI, age or gender.

To further validate the bacterial alterations in HTN, an independent metagenomic analysis was performed using the sequencing data generated from a previous study of type 2 diabetes [2]. From a total of 174 non-diabetic controls in the study, normotensive controls with SBP ≤125 mmHg or DBP ≤80 mmHg were enrolled, and HTN were elected with the inclusion criteria of SBP ≥150 mmHg or DBP ≥100 mmHg. The FBG levels between normotensive controls and HTN were similar. Finally, six subjects (HTNs, *n* = 3; normotensive controls, *n* = 3) were included in our analysis (Additional file 2: Table S6). As expected, the microbial diversity was decreased in HTN (Additional file 6: Figure S4a), and there were at least 20 genera showing consistent trends with our findings, including decreased *Butyrivibrio*, *Clostridium*, *Faecalibacterium*, *Enterococcus*, *Roseburia*, *Blautia*, *Oscillbacter*, and elevated *Klebsiella*, *Prevotella*, and *Desulfovibrio* (Additional file 6: Figure S4b, Additional file 2: Table S7).

Collectively, these results supported our hypothesis that bacteria associated with healthy status were reduced in patients with HTN. This phenomenon together with the overgrowth of bacteria such as *Prevotella* and *Klebsiella* may play important role in the pathology of HTN.

### Co-abundance groups enriched in pHTN and HTN

Firstly, for each gene, an OR score was calculated according to the abundance of each gene. Then, for the comparative analysis between control and HTN samples, the HTN-associated genes were classified as HTN-enriched (OR >2) or HTN-depleted (OR <0.5) as previously described [34]. When calculating HTN-associated ORs, samples of pHTN were excluded, and samples labeled as HTN were excluded as well when calculating pHTN-associated ORs. A total of 1,120,526 genes significantly different in relative abundance across groups were identified (Additional file 7: Table S8). Secondly, we clustered genes by a rather high threshold (Spearman’s correlation coefficient ≥0.7) according to previous methods [4, 35]. Spearman’s correlation coefficient was analyzed by R. The cluster groups with at least 50 genes were defined as co-abundance groups (CAGs) [4], and used for further analysis [35]. One thousand ninety-nine distinct CAGs were obtained (Additional file 2: Table S9–S11 and Additional file 8: Figure S5a). Seven hundred fourteen CAGs were assigned to known bacterial genera based on the tracer genes, with at least 80% of the genes mapped to the reference genome at an identity higher than 85% (Additional file 8: Figure S5b).

**Fig. 3:**
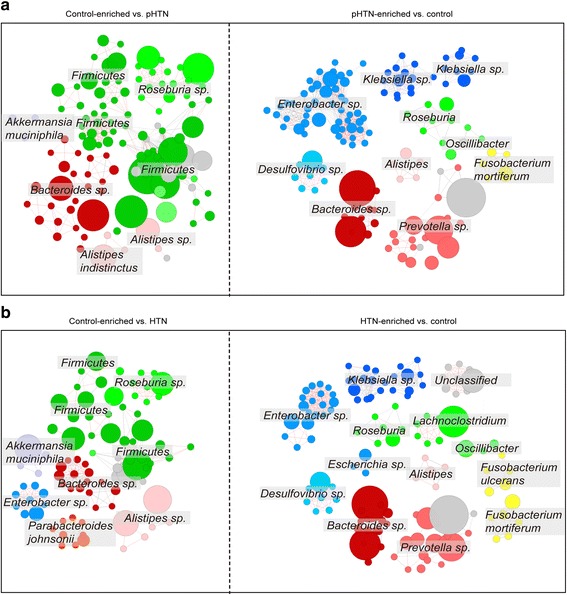
Comparative analysis of GM enrichment across groups based on CAGs. **a** CAGs are defined as a minimum of 50 linked genes, and the correlation network of CAGs differentially enriched in pHTN and the control group is performed by Spearman’s correlation based on the abundance. **b** The network of CAGs enriched in HTN is compared to controls. CAGs are colored according to the taxonomic assignment as labeled, and the node size is scaled with the number of genes within the CAG. Edges between nodes denote Spearman correlation >0.8 (*red*) or between 0.7 and 0.8 (*gray*)

CAGs were further clustered by Spearman’s correlation based on the abundance. Compared with the controls, there were 316 CAGs and 372 CAGs enriched in pHTN and HTN, respectively (Additional file 2: Table S12). In the control group, *Firmicutes* and *Roseburia* were more abundant (Fig. 3a, b). Most CAGs enriched in pre-hypertensive samples were originated from *Enterobacter*, a disease-causing bacteria linked to obesity. *Klebsiella*, causally implicated in various infections, was also overrepresented in pre-hypertensive and hypertensive patients [27]. Additionally, most recent studies revealed that *Fusobacterium* was enriched in the fecal samples of patients with liver cirrhosis, colorectal carcinoma, or ulcerative colitis [4, 36, 37]. We also detected several clusters of CAGs assigned to *Fusobacterium* enriched in pHTN and HTN groups. About 200 CAGs were different in pHTN and HTN. Most of them in pHTN were from *Enterobacter* and *Klebsiella*, while *Prevotella* and *Fusobacterium* were more abundant in HTN.

To further examine the relationship between clinical indices and CAGs of GM, physiological parameters of SBP, DBP, BMI, FBG, total cholesterol (TC), triglyceride (TG), and low-density lipoprotein (LDL) were included in a Spearman’s correlation analysis. We observed that SBP and DBP could negatively influence the CAGs enriched in the control group, such as *Firmicutes* and *Roseburia*, and positively interacted with *Prevotella* and *Desulfovibrio*, which were abundant in pHTN and HTN (Additional file 9: Figure S6). Whereas, both TC and TG were negatively correlated with *Enterobacter*, that was enriched in pHTN and HTN groups. Altogether, these results indicated that the bacterial communities in individuals with pHTN and HTN are similar, and the collective effect of these bacteria may account for intestinal dysbiosis in HTN.

### Functional alteration in GM of pHTN and HTN

**Fig. 4:**
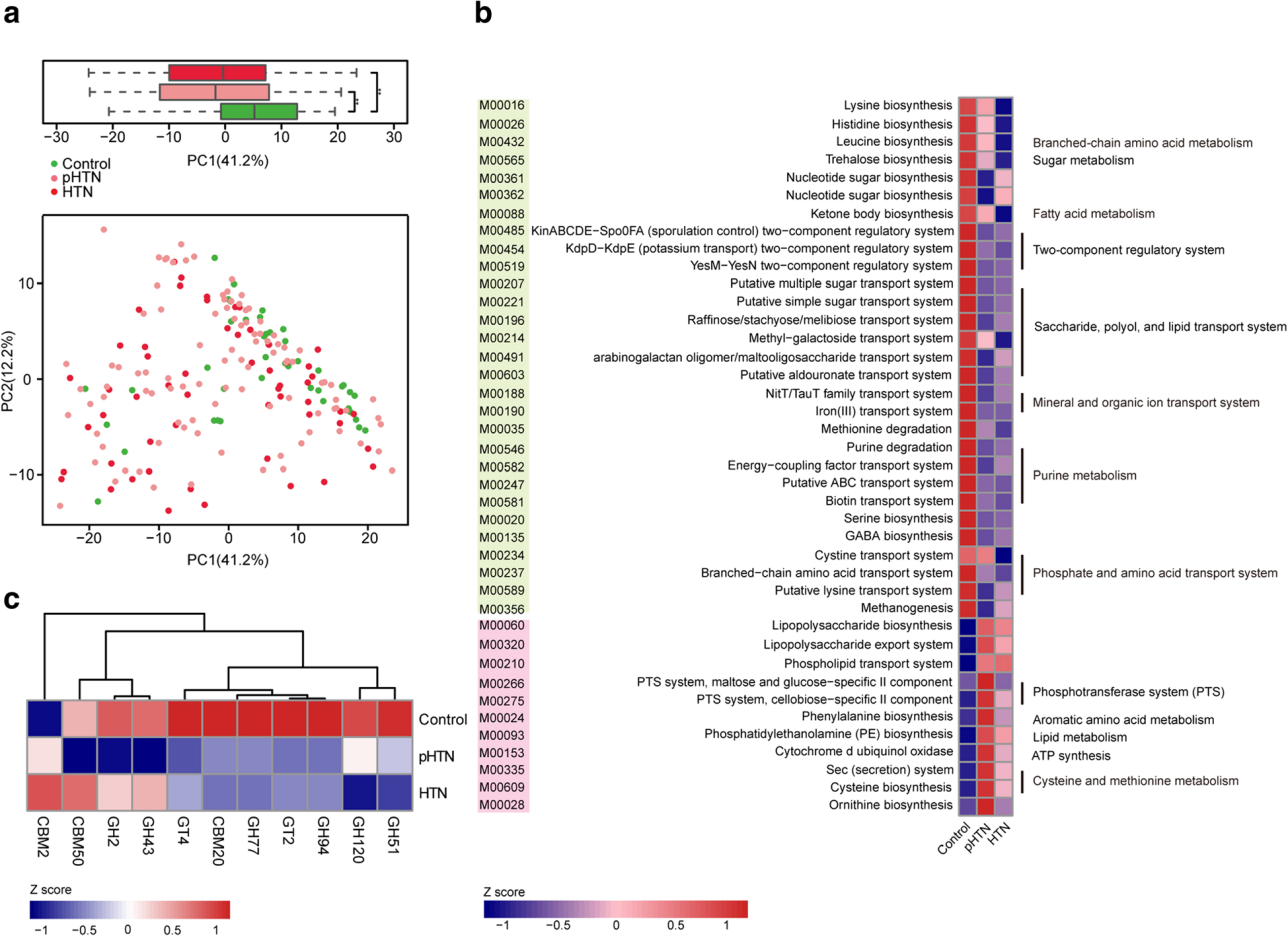
Microbial gene functions annotation in pHTN and HTN. **a** PCA based on the relative abundance of KEGG orthology groups in 196 samples. Significant differences across groups are established at the first principal component (PC1) values, and shown in the *box plots above*. **P value <0.001, Wilcoxon rank sum test. **b** The average abundance of KEGG modules differentially enriched in control, pHTN, and HTN gut microbiome. Twenty nine modules enriched in control, and 11 modules overrepresented in both pHTN and HTN are shown in *green* and *pink*, respectively. The functional potential of KEGG modules are demonstrated on the *right*. **c** Heat map showing the abundance of 11 most significantly altered CAZy family in pHTN or HTN as compared to control

Using the Kyoto Encyclopedia of Genes and Genomes (KEGG) and Carbohydrate-Active EnZymes (CAZy) [38] database, we evaluated gut microbial functions across groups in our study cohort. All the genes were aligned to the KEGG database and CAZy database, and proteins were assigned to the KEGG orthology and CAZy families (Additional file 2: Table S13–S15). Principal component analysis (PCA) based on KEGG orthology revealed striking differences in microbial functions at the first principal component (PC1) between controls and patients (*P* < 0.001, Wilcoxon rank sum test, Fig. 4a). Nearly all the KEGG modules and CAZy families displayed a similar discrepancy in pHTN and HTN when compared with the controls (Fig. 4b, c), illustrating the common functional features in pHTN and HTN. Sixty-five (*n* = 65) KEGG modules were differentially enriched among the three groups (adjusted *P* value <0.05, Wilcoxon rank sum test, Additional file 2: Table S12). The thirty-nine (*n* = 39) modules decreased in pHTN and HTN groups were involved in branched-chain amino acid biosynthesis and transport, ketone body biosynthesis, two-component regulatory system, and degradation of methionine and purine (Fig. 4b). These metabolic functions are essential for the host and have been observed in healthy populations [4, 5, 39, 40]. Although previous studies have found that iron, phosphate, and amino acid transport system, GABA biosynthesis, and methanogenesis were enriched in the patients subjected to colorectal cancer or liver cirrhosis [4, 39], these metabolic functions were not enriched in our patient cohort. We observed seventeen (*n* = 17) modules elevated in pHTN and HTN, including LPS biosynthesis and export, phospholipid transport, phosphotransferase system (PTS), biosynthesis of phenylalanine and phosphatidylethanolamine, and secretion system (Fig. 4b). The capacity to synthesize and export LPS of the gut microbiome in patients with colorectal carcinoma has been suggested to represent an important mechanism whereby inflammation contributes to tumor progression [5, 41, 42]. PTS system, phosphatidylethanolamine biosynthesis, secretion system, and transport of phospholipid, which were overrepresented in pHTN and HTN, are also linked to diabetes, liver cirrhosis, and rheumatoid arthritis [2, 4]. Additionally, the metagenome of patients were enriched in genes associated with cellulose-binding domains but depleted in host glycan-utilizing enzymes (Fig. 4c). These gut microbial functions in hypertensive patients are commonly associated with other diseases. Although the functional annotation analyses are predictive, it indicated that impairment of GM may evoke a disease-linked state through interference of physiological metabolic functions.

### Metabolic profiling of GM in pHTN and HTN

**Fig. 5:**
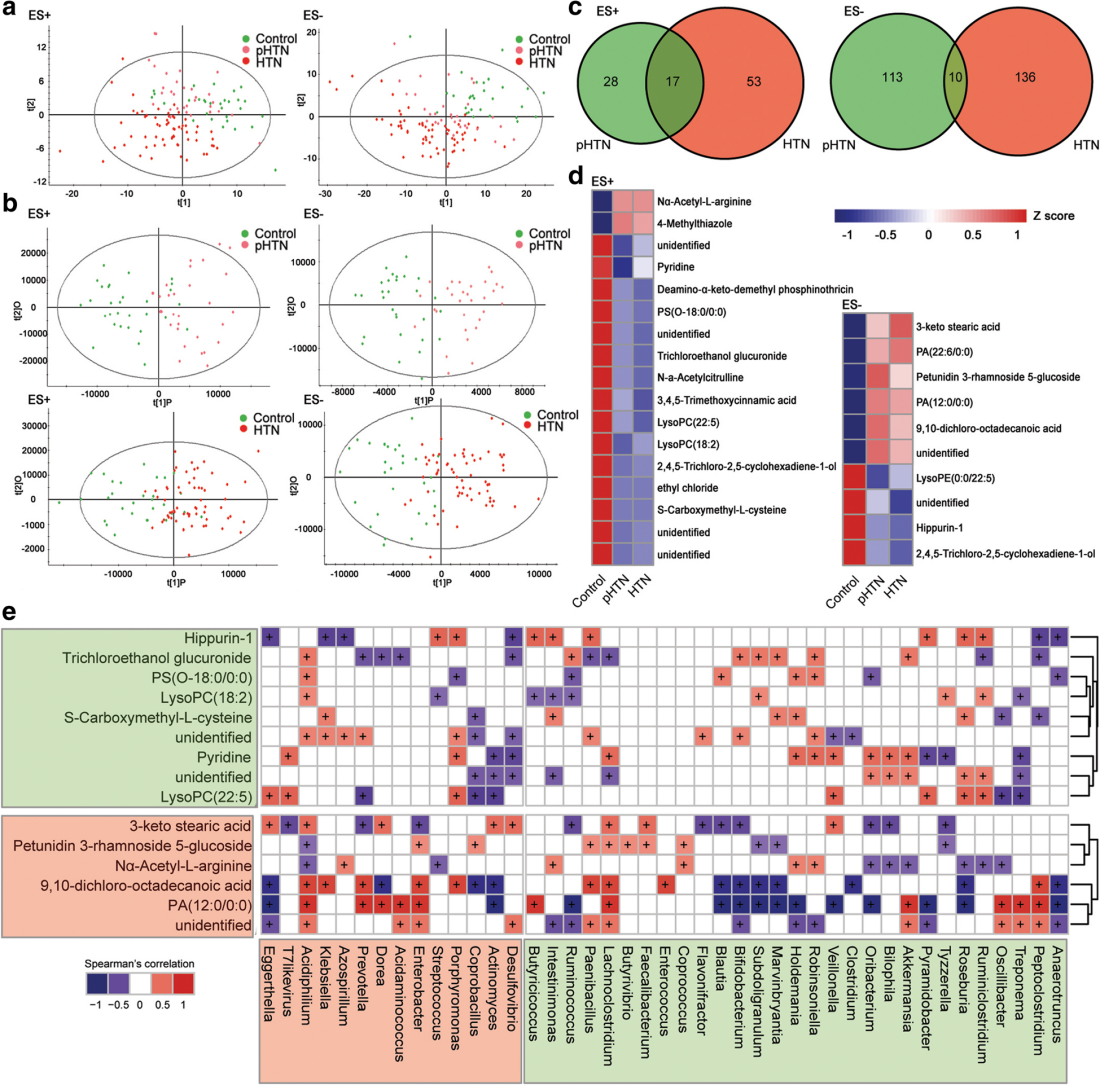
Aberrant metabolic patterns in pHTN and HTN. **a** PLS-DA score plots based on the metabolic profiles in serum samples from control, pHTN, and HTN group in ES+ and ES−. *n* = 30 for control, *n* = 31 for pHTN, and *n* = 63 for HTN. **b** Score scatter plots of OPLS-DA comparing the metabolic differences identify the separation between pHTN and control, HTN and control, respectively. **c** Metabolites significantly changed in pHTN or HTN as compared to control at VIP >1.5 and *P* value (*t* test) <0.05 are identified. Venn diagrams demonstrate the number of altered metabolites shared between pHTN (*green*) and HTN (*red*) by the overlap. **d** The relative amount of 26 endogenous compounds concurrently varied in both pHTN and HTN groups is transformed into Z scores in the heat map. There are six metabolites failed to be identified. **e** The relationship between 26 endogenous metabolites and the 44 top altered genera (Fig. 2a) in pHTN and HTN is estimated by Spearman’s correlation analysis. And those with low correlated (|*r*| <0.4) are not shown. Genera and metabolites are distinguished as abundant in control (*green*) or HTN (*pink*)

Considering the aberrant function profiles of gut microbes in disease subjects, we wondered the microbe-host interactions in HTN. As some end products of fermentation by the GM could enter the bloodstream and exert important influences on the physiology of the hosts, we explored the host metabolic profiling in fasting serum of a subset of 124 subjects by high-throughput liquid chromatography-mass spectrometry (LC/MS) and examined the relationship between GM and metabolites in the circulation. Thirty healthy controls, 31 pHTNs, and 63 patients of HTN from our previous cohort were randomly enrolled. The serum samples were subjected to LC/MS analysis in both positive ion mode (ES+) and negative ion mode (ES−). After eliminating the impurity peaks and duplicate identifications, we identified a total of 1290 chromatographic peaks in ES+ and 2289 variables in ES− for further analyses. To discriminate the metabolic profiles across groups, we performed clustering analyses based on partial least-squares discriminant analysis (PLS-DA) and orthogonal partial least-squares discriminant analysis (OPLS-DA). The serum samples from distinct groups were largely separated according to the PLS-DA plots (Fig. 5a). The scatter plots in pHTN group were closer to those in HTN, suggesting a similar metabolic mode. Furthermore, individuals in either pHTN or HTN groups were separated from the controls as further evidenced by the OPLS-DA score scatter plots (Fig. 5b).

The compositional changes in patients involved 167 analytes that were significantly different between pHTN and control, and 215 analytes altered in HTN (Fig. 5c). There were 26 metabolites which were obviously different in both pHTN and HTN groups as compared to the control (Additional file 2: Table S16). Notably, these metabolites exhibited statistically analogous profiles of alterations in pHTN and HTN, which was consistent with our findings based on gut microbiome (Fig. 5d). Endogenous compounds whose levels significantly decreased in pHTN and HTN include phosphatidylserine (PS), 3,4,5-trimethoxycinnamic acid, lysophosphatidylcholine (LysoPC), S-carboxymethyl-l-cysteine, and lysophosphatidylethanolamine (LysoPE). 3,4,5-Trimethoxycinnamic acid is capable to protect against inflammatory diseases through suppressing cell adhesion molecules in vascular endothelial cells [43]. Also S-Carboxymethyl-l-cysteine exerts anti-inflammatory properties [44]. These observed downregulations could promote the inflammatory environment associated with HTN. On the other hand, endogenous compounds whose levels significantly increased in pHTN and HTN include metabolites such as N*α*-acetyl-l-arginine, stearic acid, phosphatidic acid (PA), and glucoside. Elevated levels of Nα-acetyl-l-arginine and stearic acid have been previously observed in uremia and spontaneously hypertensive rats [45, 46]. These compounds may represent possible markers for the development of HTN and might be derived from gut microflora or their fermented products. To explore this idea, the relationship between 26 representative metabolites and the 44 most different genera was examined by correlation analysis (Fig. 5e). Control-enriched trichloroethanol glucuronide was positively correlated with *Bifidobacterium* and *Akkermansia*, but negatively linked to *Prevotella*. Conversely, there was a positive association between 9,10-dichloro-octadecanoic acid (stearic acid) and microflora including *Klebsiella*, *Prevotella*, and *Enterbacter*, which were all overrepresented in HTN. It was accordant that both *Bifidobacterium* and *Roseburia* negatively interacted with 9,10-dichloro-octadecanoic acid, which was hence considered as an important GM-influenced metabolic product in HTN. Thus the distinguished metabolic profiling in HTN was closely connected to intestinal microflora variation, although whether these metabolic products were directly metabolized by the intestinal microorganisms remained to be explored.

### Identification of pHTN and HTN basing on gut microbiome

To illustrate the microbial and metabolic signature of pHTN and HTN, and further exploit the potential of gut microbiome and metabolites in HTN identification, random forest disease classifier using explanatory variables of CAGs, metabolites, and species abundances were performed. Tenfold cross-validation was repeated for five times and the receiver operating characteristic (ROC) curves for classifying pHTN and HTN patients from controls were developed.

**Fig. 6:**
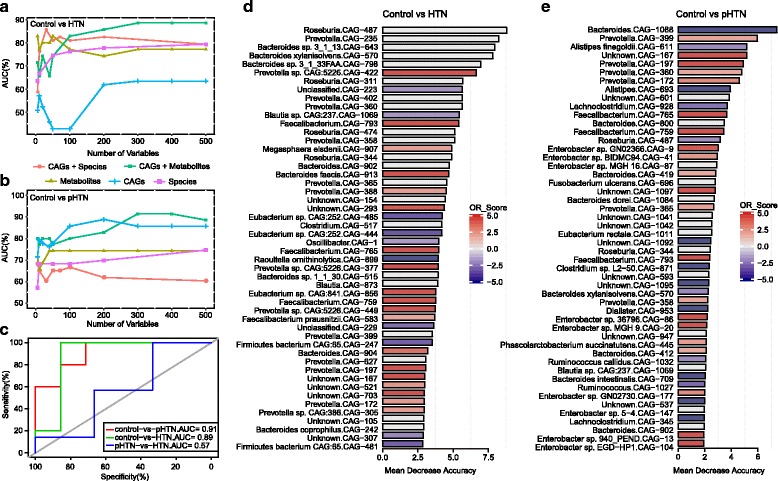
A classification to identify pHTN and HTN patients from controls. **a**, **b** Random forest models are constructed using explanatory variables of CAGs + species (*red curve*), CAGs + metabolites (*green curve*), metabolites (*yellow curve*), CAGs (*blue curve*), and species (*purple curve*). The AUC shows the classification of control versus HTN, or control versus pHTN as the numbers of variables increase. The CAGs + metabolites-based classification is more efficient as indicated by a higher AUC. **c** ROC of the random forest classifier using CAGs + metabolites based on the 1000 most important variables by ranking the variables by importance. AUC = 0.91 for control versus pHTN (*n* = 12, *red curve*), AUC = 0.89 for control versus HTN (*n* = 12, *green curve*), and AUC = 0.57 for pHTN versus HTN (*n* = 13, *blue curve*). **d** The top 50 different CAGs distinguish HTN from control based on the random forest model using explanatory variables of CAGs + metabolites. **e** The top 50 CAGs discriminate between pHTN and control using explanatory variables of CAGs + metabolites. The lengths of bar in the histogram represent mean decrease accuracy, which indicates the importance of the CAG for classification. The color denote the enrichment of CAG in control (*blue*), in HTN or pHTN (*red*) according to OR score

We could detect HTN individuals accurately based on the gut CAGs + metabolites, as indicated by the area under the receiver operating curve (AUC) of up to 0.91, and 95% confidence interval (CI) of 0.75–1 (Fig. 6a). Similarly, comparing to the other variables, the variable of CAGs + metabolites was more effective to classify pHTN samples from controls, showing an AUC of 0.89, and 95% CI of 0.65–1 (Fig. 6b). Thus, we conducted a testing set consisted of 13 randomly chosen subjects based on CAGs + metabolites. In this assessment analysis, both pHTN and HTN patients possess remarkable features in gut microbiome and metabolites as compared to the controls (Fig. 6c). However, we observed poor performance on the test set when discriminating between pHTN and HTN by lower specificity and sensitivity (AUC, 0.57; 95% CI, 0.21–0.93; Fig. 6c). This further validated the similarity of pHTN and HTN in our previous findings. Among the most discriminatory CAGs to distinguish pHTN or HTN from control, there were some CAGs similarly enriched in both pHTN and HTN subjects, including CAG-172 (*Prevotella*), CAG-197 (*Prevotella*), CAG-759 (*Faecalibacterium*), CAG-765 (*Faecalibacterium*), and CAG-793 (*Faecalibacterium*) (Fig. 6d). These CAG markers were the common microbial characteristics of pHTN and HTN and contributed a lot to the identification of pHTN and HTN.

We also investigated the utility of the classifier based on microbial CAGs + species. Consistently, the AUC for identifying pHTN and HTN from the controls was 0.67 (95% CI, 0.39–0.95) and 0.81 (95% CI, 0.53–1), respectively, and the performance on pHTN and HTN individuals was not as satisfactory (AUC, 0.47; 95% CI, 0.19–0.75; Additional file 10: Figure S7a). For HTN classification, CAGs and species taxonomic annotated to *Prevotella*, including Prevotella sp. CAG:5226.CAG-377, *Prevotella bivia*, and CAG-184 were typically important (Additional file 10: Figure S7b). Overall, the pHTN- and HTN-associated microbial and metabolic features captured by the classifier offered further evidence for dysbiotic gut microbiome and highlighted great potential ability for detection of pHTN and HTN populations by GM and metabolites.

### High BP is transferrable by fecal transplant

Previous studies have revealed that antibiotics and probiotics are potential treatment modalities for BP in both animal models and clinical trials [19, 21, 22, 47]. We speculated that the alterations in GM under pro/antibiotic use may be associated with BP changes. There is evidence that Dahl salt-sensitive rats transplanted with salt-resistant rat microbiota have further exacerbated BP, which indicate that the microbiota resident within the cecum of the Dahl salt-sensitive rat, but not the salt-resistant rat, are in a symbiotic relationship with the host [20]. Thus the differences between Dahl salt-sensitive rats and the salt-resistant rats are highlighted. Investigators have also proved that transplantation of cecal contents from hypertensive obstructive sleep apnea rats on high-fat diet into the same obstructive sleep apnea recipient rats on normal chow diet lead to higher BP similar to the donors [23]. In this study, it seems that a major contributor to the gut dysbiosis of HTN is a high-fat diet. Therefore, direct studies testing if microbial transplantation can transmit changes in BP from hypertensive donors to recipients are still lacking. To further demonstrate whether alterations of GM are a causal factor for the progression of HTN in vivo, fecal bacteria from hypertensive patients were transplanted to GF mice in the present work.

**Fig. 7:**
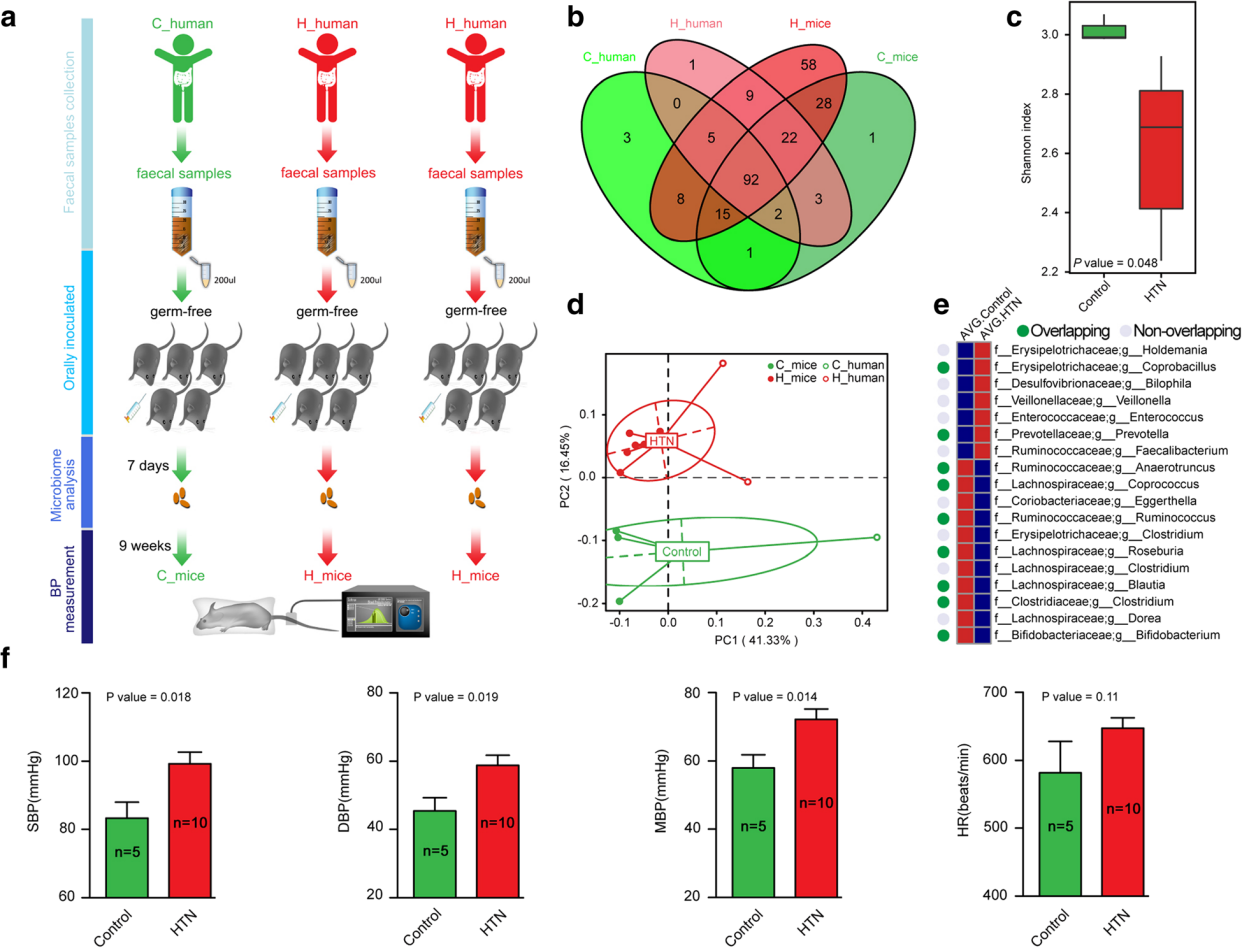
Post-transplanted intestinal microbial profiles and BP of recipient mice. **a** Schematic representation of fecal microbiota transplantation. GF mice (*n* = 5 for control, *n* = 10 for HTN) are orally inoculated with prepared fecal contents from two patients of HTN and one normotensive control, respectively. The gut microbial profiles are analyzed at 7 days, and BP is measured at 10 weeks post-transplantation. C, control; H, HTN. **b** Venn diagram comparing the shared genera number in gut microbiome of human donors (*n* = 1 for control, *n* = 2 for HTN) and recipient mice (*n* = 3 for control, *n* = 6 for HTN). **c** Shannon index of recipient mice at the genus level demonstrate significantly reduced *α* diversity in HTN group. *P* = 0.048 from *t* test. *Boxes* represent the inter quartile ranges, *lines* inside the boxes denote medians, and *circles* are outliers. **d** PCoA plots of human donors and recipient mice based on microbial genera separate HTN group from the controls. **e** Heat map comparing the abundance of altered genera between control and HTN mice. *Red*, more abundant; *blue*, less abundant. Genera present consistent trend with the metagenomic analysis are marked with *green points*, while inconsistent with *gray points*. **f** SBP, DBP, MBP, and HR of the recipient mice (*n* = 5 for control, *n* = 10 for HTN) are measured by tail-cuff method. Data are presented as mean ± s.e.m. *P* = 0.018, SBP; *P* = 0.019, DBP; *P* = 0.014, MBP; *P* = 0.11, HR; *t* test

The donors for microbiota transplantation consisted of two patients of HTN and one normotensive control (Additional file 11: Table S17). They were strictly selected, and fresh fecal samples from donors were inoculated to recipient mice as soon as possible. Male GF mice at 8–10 weeks were divided into groups and orally inoculated with stool samples two times at 1-day interval (Fig. 7a). The fecal samples of recipient mice post-transplantation were investigated by 16S V4 region amplicon sequencing (Additional file 2: Table S18). The sequences were de novo clustered at 97% sequence identity and annotated to genera. From HTN patients, 128 genera were successfully colonized in the intestine of HTN mice, and 110 genera were transferred to control-mice from the control donor (Fig. 7b). Shannon index based on the genera profile showed reduced bacterial diversity in HTN mice (P = 0.048; *t* test, Fig. 7c). As expected, PCoA at the genus level clustered HTN patients and mice colonized with hypertensive GM into one group, but control and control mice into a separated group (Fig. 7d). Moreover, at the genus level, *Anaerotruncus*, *Coprococcus*, *Ruminococcus*, *Clostridium*, *Roseburia*, *Blautia*, and *Bifidobasterium* were confirmed to be deficient, while *Coprobacillus* and *Prevotella* were shown to be more abundant in HTN mice, which was in agreement with our previous observations in the metagenomic analyses (Additional file 2: Table S19, Fig. 7e).

At 10 weeks post-transplantation, BP of recipient mice in HTN and control group was measured by the tail-cuff method. Notably, the HTN mice exhibited significantly higher SBP, DBP, and mean blood pressure (MBP) as compared to controls (*P* < 0.05), as well as elevated heart rate (*P* = 0.11) (Fig. 7f). Early studies have shown that when compared to conventional controls, GF rats possess significantly lower cardiac output, relatively diminished regional blood flow, lower level of systemic BP response after blood loss, and hypotonic microvasculature [48], which might lead to a low systolic BP in the recipient mice. These findings provided novel and direct evidence that GM could influence the BP of host directly. Therefore, changes in the GM might be the mechanism underlying the effect of antibiotics and probiotics on BP control. As the number of donors for transplantation is limited, larger number of fecal transplants from hypertensive, pre-hypertensive, and normotensive control participants should be carried out in the future to further establish the magnitude of BP changes.

## Discussion

To date, there are limited studies indicating a direct association between GM and HTN, especially in human disease. Several important gaps in knowledge of gut and BP remain unexplored, and critical issues should be addressed, such as the microbial profiles of HTN populations in clinical trials, the metabolites signature profiles, the microbial biomarkers for early detection of HTN, and fecal transplantation to make clear the causal relationship between gut dysbiosis and HTN. To make up for these blanks, we applied a strategy based on metagenomic and metabolomic analyses, coupled with GM transplantation. We sequenced the total bacteria DNA of stool samples from a cohort of 196 Chinese individuals and supplemented this analysis with an additional validation cohort. All the individuals in the present study are from a cohort study among employees of the Kailuan Group Corporation. The Kailuan study is a prospective cohort study focusing on the Kailuan community in Tangshan, a large modern city in northern China. As the subjects were from a relatively concentrated environment, the differences in the diets were relatively small. All the subjects in the hypertension group were newly diagnosed hypertensive patients prior to antihypertensive treatment. Patients suffering from cancer, heart failure, renal failure, smoking, stroke, peripheral artery disease, and chronic inflammatory disease were all excluded. Drugs including statins, aspirin, insulin, metformin, nifedipine, and metoprolol were not used on the patients, and other drug consumption was not compared because the sample size was quite small. Hence, it is not likely that the medication use directly influenced the gut metagenome and metabolites, as there was no significant difference in the drugs consumed by these subjects. Our results demonstrate that decreased diversity, altered enterotype distribution, and variation in bacteria populations were associated with both pHTN and HTN. The bacterial metabolic functions and GM-related metabolites in pre-hypertensive and hypertensive adults were closely linked to inflammatory state. Particularly, both pHTN and HTN individuals could be accurately distinguished from the controls by variables of CAGs and metabolites. And most importantly, the direct impact of GM composition on regulating BP was evaluated using an in vivo model of GF mice colonized with human intestinal microbiota. Bacteria such as *Prevotella*, *Klebsiella*, *Enterobacter*, and *Fusobacterium* are potential candidates for further bacteria transfer experiments to explore the precise mechanisms underlying the effect of GM in BP regulation. Our work provides the first direct evidence that highlights the pivotal role of dysbiotic gut microbiome as an important pathogenic factor for the high BP of the host. Thus GM modulation should be considered during antihypertensive treatment.

Researchers previously suggested that the intestinal bacterium *Prevotella copri* thrives in a pro-inflammatory environment of rheumatoid arthritis [3, 49]. The superoxide reductase and phosphoadenosine phosphosulphate reductase encoded by *Prevotella copri* may favor the development of inflammation. In their further demonstration, colonization with *Prevotella copri* enhances body weight loss and exacerbates epithelial inflammation in colitis mouse model [3]. Interestingly, as shown by our data, the enterotype dominated by *Prevotella* was enriched with pHTN and HTN populations. Moreover, *Prevotella* was overrepresented in individuals with pHTN and HTN. And stearic acid, an important metabolite in HTN, was positively linked to *Prevotella*. Furthermore, CAGs and species taxonomic annotated to *Prevotella* were the common microbial characteristics of pHTN and HTN, and contributed a lot to classification of HTN. Thus *Prevotella* may play an essential role in HTN, probably by triggering the inflammatory response. Our findings have consolidated the potential of *Prevotella* in the pathogenesis of diseases, and call for further exploring whether *Prevotella* is a causal conducer to inflammation and HTN.

Concomitant with the alteration of gut microbial composition, we observed a dysbiosis in bacterial gene functions. The metagenome of HTN patients were depleted in genes associated with biosynthesis and transport of amino acid, such as lysine, histidine, leucine, and serine, which are essential for human health. Functional annotation also indicated a decline of modules for fatty acid utilization and saccharide transport, suggesting an impaired capacity of energy production. In agreement with previous studies showing a dearth of microbial functions for purine metabolism in arthritis [3], a significant decrease in purine-metabolizing enzymes was identified to be related to HTN. Indeed, these metabolic functions are quite necessary for healthy populations [3–5, 39, 40]. In contrast, the enrichment of the modules for LPS biosynthesis and export in patients hints at a potential role of GM in causing low-grade inflammation. Inflammation due to immune response triggered by LPS is the cardinal feature of the pathogenesis of gram-negative bacteria, such as *Prevotella* and *Klebsiella* [50, 51], and has been identified as an important contributor to the pathogenesis of HTN. Our findings raise the possibility that the low-grade inflammation and increase of gram-negative bacteria, especially *Prevotella* and *Klebsiella*, are likely responsible for HTN pathology. Thus, our analysis of bacterial gene functions indicates that functional dysbiosis may contribute to the susceptibility to HTN, and overproduction of LPS by gut bacteria seems to be directly linked to HTN development, whereas amino acid biosynthesis, fatty acid utilization, and purine metabolism by bacteria might have a role in HTN prevention.

Actually, in GF mice, the tail-cuff method has been used for assessment of BP in a recent report, suggesting the methodology is acceptable [52]. In our study, the tail-cuff measurement was performed as the others did previously, and indicated a tendency for higher BP in recipient mice inoculated with stool samples from hypertensive donors as compared to controls. As such results were not obtained by fecal microbiota transplantation in conventionally raised mice, we speculate that the immune inflammatory system might play a crucial role in the pathogenesis of HTN. Further mechanism research to make clear whether gut bacterial metabolites show a contribution to the immune inflammatory system during the development of HTN is being performed.

In HTN studies, most work focused on patients with a clinical definition of HTN, who display a SBP higher than 140 mmHg or DBP ≥90 mmHg. However, population studies suggest that there is an intermediate stage of BP between control and HTN defined as pHTN, which should not be ignored. In our study, we considered subjects with pHTN as an independent group. Surprisingly, the bacterial diversity, enterotype, composition, and metabolic functions, as well as classified characteristics in pHTN highly coincided with those in HTN. As shown in Figs. 1, 2, and 3, there was a little difference in the structure of gut microbiome between pHTN and HTN, indicating that pHTN is not simply a transition stage between normotensive and hypertensive status upon BP levels but rather a state in which gut dysbiosis has already occurred. Moreover, our findings revealed indiscriminate metabolic profilings between pHTN and HTN, consistent with a previous report that the serum spectral profiles of the hosts were similar at a stage of SBP ≥130 mmHg and at SBP ≥150 mmHg [53]. The close correlation of metabolic products and GM further strengthened and highlighted the importance of pHTN. Therefore, early treatment of pHTN has strong clinical value. In agreement with our notion, high BP has become one of the three leading risk factors for death according to the Global Burden of Disease Study [54]. Moreover, our findings fully support the updated conclusion by the Systolic Blood Pressure Intervention Trial (SPRINT) research group, that controlling one’s SBP to an optimal level lower than 120 mmHg rather than a pHTN level below 140 mmHg leads to significantly decreased occurrence of cardiovascular events and death [55]. Thus, more attention should be given to the previously neglected populations in pHTN, and early intervention for pHTN is strongly appealed.

## Conclusions

Taken together, we have described clearly the disordered profiles of GM and microbial products in human patients with pHTN and HTN, established the relationship between gut dysbiosis and HTN, and provided important evidence for the novel role of GM dysbiosis as a key factor for BP changes. Our findings point towards a new strategy aimed at preventing the development of HTN and reducing cardiovascular risks through restoring the homeostasis of GM, by improving diet and lifestyle or early intervening with drugs or probiotics.

## Methods

### Study cohort and patient characteristics

All the individuals in the present study were from a cohort study among employees of the Kailuan Group Corporation. The Kailuan study is a prospective cohort study focusing on the Kailuan community in Tangshan, a large modern city in northern China, where 11 hospitals are responsible for the health care of the community, all of which participated in conducting physical examinations. All the subjects in the current work were strictly enrolled and none of them was under antihypertensive treatment. The participants were classified based on the Internal Guidelines for HTN as described in Additional file 1: Table S1. It was composed of 41 healthy controls (SBP ≤125 mmHg, or DBP ≤80 mmHg), 56 pHTNs (125 mmHg < SBP ≤ 139 mmHg, or 80 mmHg < DBP ≤ 89 mmHg), 99 patients of HTN (140 mmHg ≤ SBP, or 90 mmHg ≤ DBP). BP was measured in a sitting position by nurses or physicians. Three readings were recorded at 5-min intervals with a random-zero mercury column sphygmomanometer, and the average was taken as the final measurement.

All clinical information was collected according to standard procedures. Patients suffering from cancer, heart failure, renal failure, smoking, stroke, and peripheral artery disease were excluded, and none of the patients was under antihypertensive treatment. Healthy volunteers had no history of diabetes mellitus or hypercholesterolemia. Individuals were also excluded if they had received antibiotics or probiotics within the last 8 weeks. The study was approved by local ethics committees (Kailuan General Hospital, Beijing Chaoyang Hospital, and Beijing Fuwai Hospital) and informed consent was obtained from all subjects.

### Stool sample collection and DNA extraction

Stool samples freshly collected from each participant were immediately frozen at −20 °C and transported to the laboratory with ice pack. Bacterial DNA was extracted at Novogene Bioinformatics Technology Co., Ltd using TIANGEN kit according to the manufacturer’s recommendations.

### Metagenomic sequencing and gene catalogue construction

All samples were paired-end sequenced on the Illumina platform (insert size 300 bp, read length 125 bp) at the Novogene Bioinformatics Technology Co., Ltd. After quality control, the reads aligned to the human genome (alignment with SOAP2 [56], Version 2.21, parameters: -s 135, -l 30, -v 7,-m 200,-x 400) were removed. The remaining high-quality reads were used for further analysis.

The assembly of reads was executed using SOAP denovo (Version 2.04, parameters: -d 1 -M 3 -R -u -F) [57]. For each sample, we used a series of k-mer values (from 49 to 87) and chose the optimal one with the longest N50 value for the remaining scaffolds [4]. We mapped the clean data against scaffolds using SOAP2 (Version 2.21, parameters: -m 200 -x 400 -s 119). Unused reads from each sample were assembled using the same parameters. Genes (minimum length of 100 nucleotides) were predicted on scaftigs (i.e., continuous sequences within scaffolds) longer than 500 bp using MetaGeneMark (prokaryotic GeneMark.hmm version 2.10). Then, a non-redundant gene catalogue was constructed with CD-HIT (version 4.5.8, parameters: -G 0 -aS 0.9 -g 1 -d 0 -c 0.95) [58] using a sequence identity cut-off of 0.95, with a minimum coverage cut-off of 0.9 for the shorter sequences.

To determine the abundance of genes, reads were realigned to the gene catalogue with SOAP2 using parameters: -m 200 -x 400 -s 119. Only genes with ≥2 mapped reads were deemed to be present in a sample [59]. The abundance of genes was calculated by counting the number of reads and normalizing by gene length.

### *α* diversity and rarefaction curve

To estimate the genera richness of the sample, we calculated the within-sample (*α*) diversity using Shannon index based on the genera profiles. A high *α* diversity indicates a high richness of genera within the sample.

Rarefaction analysis was performed to assess the gene richness in the controls, pHTN, and HTN. For a given number of samples, we performed random sampling 100 times in the cohort with replacement and estimated the total number of genes that could be identified from these samples by R (Version 2.15.3, vegan package).

### Microbial community types (enterotypes)

The community types of each sample were analyzed by the PAM method using relative abundance of genera. The optimal number of clusters was estimated using the CH index, as previously described [60]. Only genera with an average relative abundance ≥10^−4^ and existed in at least six samples were considered in the analysis. According to Spearman’s correlation between genera abundances, the genera in enterotype 1 were clustered, and the co-occurrence network of them was visualized by Cytoscape (Version 3.2.1).

### Taxonomic annotation and abundance profiling

To assess the taxonomic assignment, genes were aligned to the integrated NR database using DIAMOND (Version 0.7.9.58, default parameter except that -k 50 -sensitive -e 0.00001) [61]. As previously described [59], for each gene, the significant matches, which were defined by e-values ≤10 × e-value of the top hit, and these retained matches were used to distinguish taxonomic groups. The taxonomical level of each gene was determined by the lowest common ancestor-based algorithm and implemented in MEGAN [62]. The abundance of a taxonomic group was calculated by summing the abundance of genes annotated to a feature.

### Metagenomic analysis in the verification phase

All phenotype information of participants were listed in the supplementary tables of Qin J et. al [2]. Subjects with diabetes were excluded. Three HTN patients with SBP ≥150 mmHg or DBP ≥100 mmHg were enrolled, and three normotensive controls with SBP ≤125 mmHg and DBP ≤80 mmHg were included for the analysis.

### Co-abundance gene groups

To identify the marker genes associated with pHTN and HTN, the abundance of each gene across groups was compared according to Greenblum S et al. [34]. As previously described [35], these marker genes were clustered into groups based on their abundance variation across groups. Clusters with more than 50 genes were defined as co-abundance gene groups (CAGs), and used for further analysis. CAG abundance profiles were calculated by the average gene depth signal and weighted by gene length.

Taxonomic assignment of the CAGs was performed according to the taxonomy of tracer genes, as previously described [2]. Briefly, assignment to species requires 90% of the genes in a CAG to align with the species’ genome with 95% identity and 70% overlap of query. Assigning CAG to a genus requires 80% of its genes to align to the genome with 85% identity in both DNA and protein sequences.

### Co-occurrence network of CAGs

The enriched CAGs were identified according to Greenblum S et al.. These CAGs were clustered according to Spearman’s correlation. The co-occurrence network was visualized using Cytoscape (Version 3.2.1). The enriched CAGs/genes were identified according to Greenblum S et al. [34]. Briefly, for each CAG, an OR score was calculated according to the abundance in the set of compared samples. Then, for the comparative analysis between control and HTN samples, the HTN-associated CAGs were classified as HTN-enriched (OR >2) or HTN-depleted (OR <0.5). When calculating HTN-associated ORs, samples of pHTN were excluded from the analysis, and when calculating pHTN-associated ORs, samples labeled as HTN were excluded.

### Association between CAGs and clinical indices

Based on the clinical indices and enriched CAG profiles, we calculated Spearman’s correlation in all samples. The *P* values were corrected for multiple testing with Holm method by R (Version 2.15.3, psychpackage). Only 162 samples were considered in the analysis because of the clinical data missing in 34 samples.

### Functional annotation

All genes in our catalogue were aligned to the KEGG database (Release 73.1, with animal and plant genes removed) and CAZy database (http://www.cazy.org/) using DIAMOND (Version 0.7.9.58, default parameter except that -k 50 --sensitive -e 0.00001). Each protein was assigned to the KEGG orthology and CAZy families by the highest scoring annotated hits containing at least one HSP scoring over 60 bits [63]. The abundance of KEGG orthology/module was calculated by summing the abundance of genes annotated to the same feature.

### Metabonomics analysis based on LC/MS

One-hundred twenty-four (*n* = 124) individuals from our study cohort were subjected to metabonomics analysis based on the LC/MS method. This cohort was composed of 30 healthy controls, 31 pHTNs, and 63 patients of HTN. The whole blood samples were collected and separated into serum by centrifugation. Each serum samples at 100 μL were mixed with 400 μL methanol, and the mixtures were centrifuged at 12,000 rpm for 15 min at 4 °C. For LC/MS analysis, 200 μL of the supernatant was harvested.

The serum metabolic profiles were performed on a Thermo Fisher Ultimate 3000 LC system. For chromatographic separation, C18 (2.1 mm × 100 mm × 1.9 μm) reversed-phase column (Thermo Scientific, USA) preheated at 40 °C was used. A prepared serum sample of 4 μL was injected and maintained at 4 °C for analysis. The gradient conditions for metabolite elution were at 5% B for 0–1 min, 5–40% B for 1–2 min of linear gradient, 40–80% B for 2–11 min of linear gradient, and 95% B for 11–15 min. The mobile phase for positive ion mode (ES+) and negative ion mode (ES−) was composed of water with 0.1% formic acid as solvent A, and acetonitrile with 0.1% formic acid as solvent B, and the flow rate was at 300 μL/min.

For mass spectrometric assay, Orbitrap Elite mass spectrometer (Thermo Scientific, USA) equipped with ESI source was used to analyze the metabolite ions. The spray voltage was set to 3.8 kV in ES+ and 3.2 kV in ES−, the flow rate of sheath gas, aux gas, and sweep gas was 45, 15, and 1 arb, respectively. The ion source temperature was 300 °C, and the capillary temperature was 350 °C. Masses ranging from 50 to 1000 ion mass (m/z) were acquired, and the resolving power was set to 60,000.

The raw ESI data of LC/MS was converted into m/z format and analyzed for non-linear retention time (RT) alignment, peak detection, and filtration. Maximal spectrum of continuous wavelet transform was used to correct baseline and detect peak positions. Impurity peaks and duplicate identifications were eliminated. Compounds significantly different between groups were obtained at a variable influence on projection (VIP) >1.5, and *P* value of *t* test statistics <0.05 based on the peak intensities. The m/z values of these compounds were used to identify the metabolites corresponding to the featured peak in the Metlin database.

From the metabolite profile and 44 top differential genera abundance profile, Spearman’s correlation was performed to eliminate multi-collinearity and only one factor will be randomly selected from high correlated clusters (|*r*| ≥ 0.9) for further analysis. A stepwise regression of linear models was used for modeling the relationship between metabolites and related genera, from the fitting value of individual metabolites, and Spearman’s correlation between metabolic and associated genera was calculated and scaled by coefficients of each respective linear model.

### Animal experiments

GF C57BL/6L mice were obtained from Shanghai Institutes for Biological Sciences (SLAC Inc., Shanghai, China) and housed under a 12-h light–dark cycle in the gnotobiotic facilities. All mice were fed with sterile food and water ad libitum, and bacterial contamination was monitored by periodic examination of stools. For microbiota transplantation, the fresh fecal samples were collected from donors (Additional file 11: Table S17), resuspended with sterile saline, and centrifuged for supernatant. Male GF mice aged 8–10 weeks were randomly distributed into two groups and orally inoculated (200 μL for each mouse) twice at 1-day interval with prepared fecal contents from control or patients. Recipient mice transferred with microbiota were kept in different Trexler-type flexible film isolators, fed with sterile food and water, and bacterial contamination was strictly controlled. The gut microbial profiles of recipient mice were analyzed by 16S sequencing after 7 days. We chose a time point of 10 weeks post-transplantation for BP measurement. An assessment of BP was performed within 60 min after exporting the mice out of their gnotobiotic facilities, and we could not ensure prevention from bacterial contamination after the measurement; the BP at other time points during 10 weeks was not further examined. The BP was measured by the tail-cuff method and the BP-98A system (Softron, Tokyo, Japan), which was noninvasive and did not require surgery, since using direct invasive methods such as radiotelemetry techniques will immediately expose the mice to a non-sterile condition, which might impact the results. To acclimatize the mice undergoing the measurement procedures and improve measurement reliability, a heat-sterilized dark cover was transported into the germ-free mice isolator, where it was sterilized by spraying with a chlorine dioxide-based disinfectant in the isolator port. Before BP measurement, we have trained the mice by placing them in the dark cover in their sterile flexible film isolators without exporting them out at the same time for 14 days. To minimize contamination, the measurement was performed with UV-sterilized instruments under a sterile hood within 60 min after exporting the mice out of their sterile environment. All animal care and experiments were performed in accordance with the guidelines of Institutional Animal Care and Use Committee of SLAC Inc.

### 16S ribosomal RNA sequencing

16S rRNA community profiles were characterized using Illumina HiSeq sequencing of the V4 region (insert size 300 bp, read length 250 bp). Sequences were de novo clustered at 97% sequence identity and chimeras were removed using UPARSE [64]. For each representative sequence, the GreenGene Database was used to annotate taxonomic information [65].

### Statistical analysis

The Shannon index at the genera level was calculated with QIIME (Version 1.7.0). PCA was analyzed using the FactoMineR package in R software (Version 2.15.3). PCoA was performed and displayed by ade4 package, cluster packages, fpc packages, and clusterSim package in R software (Version 2.15.3). PLS-DA was performed using SIMCA-P software to cluster the sample plots across groups.

Differential abundance of genes, genera, and KO modules was tested by Wilcoxon rank sum test, and *P* values were corrected for multiple testing with the Benjamin & Hochberg method. Only genera with an average relative abundance ≥10^−4^ and existed in at least six subjects were considered in the analyses. Correlations between enriched CAGs and clinical indices were tested with Spearman’s correlation and visualized by Cytoscape (Version 3.2.1).

Using the profiles of species, CAGs, and metabolites, the samples were randomly divided into training set and test set. A random forest classifier was trained on 80% of the data and tested on the remaining 20% of our data using the random forest package in R. In order to evaluate the performance of the predictive model and get more precise curves, we used a 10-fold cross-validation within the training set. The cross-validational error curves (average of 10 test sets each) from five trials of the 10-fold cross-validation were averaged. Variable importance by mean decrease in accuracy was calculated for the random forest models using the full set of features. The number of variables was 1000 at the lowest cross-validational error. Thus, the predictive model was constructed using the 1000 most important variables, which were further applied for ROC analysis. The performance of the smaller models were measured as AUC when applied to the test set, and the confidence intervals for ROC curves were calculated using the pROC R package.

## Additional files

Additional file 1: Table S1. Characteristics of the study cohort. A total of 196 participants consisted of 41 healthy controls, 56 subjects of pHTN, and 99 patients with HTN were enrolled. (DOC 42 kb)
Additional file 2: Table S2. Data production of 196 samples in control, pHTN and HTN. Table S3. Relative abundance profile at the phylum level. Table S4. Relative abundance profile at the genus level. Table S5. Detailed information of differential genera. Table S6. Information of 6 samples in the verification phase. Table S7. Relative abundance profile at the genus level of 6 samples in the verification phase. Table S9. Reference genomes for CAG’s taxonomy assignment. Table S10. Detailed information of enriched CAGs in different groups. Table S11. Detailed information of 1099 CAGs. Table S12. Spearman’s correlation between enriched CAGs. Table S13. Detailed information of differential KEGG modules. Table S14. Detailed information of differential KEGG orthologys. Table S15. Detailed information of differential CAZy family. Table S16. Detailed information of 26 metabolites differently enriched across groups. Table S18. Data production for donors and recipient mice in microbiota transplantation. Table S19. Relative abundance profile at the genus level of donors and recipient mice. (XLSX 4171 kb)
Additional file 3: Figure S1. The number of enterotypes in our cohort is most rational at 2. Based on the PAM clustering method, a total of 196 stool samples are clustered into different numbers of community types with CH index, which shows the performance in recovering cluster numbers. The maximum CH index at two clusters (enterotypes) indicates the optimal enterotype number. (PDF 183 kb)
Additional file 4: Figure S2. The interaction network of genera in enterotype 1. (a) The main contributor in enterotype 1 is shown with yellow circle (*Prevotella*), genera shown by white circles link to it directly, and red ones indirectly. Edges between nodes in red denote Spearman’s correlation > 0.4, and correlation ≤0.4 is in blue. The width of edges is scaled by correlation index. (b) The twelve genera negatively correlated with *Prevotella* are all decreased in enterotype 1. Box plots are shown to compare the relative abundances of genera within the interaction network of enterotype 1. Ten out of twelve genera are significantly decreased in enterotype 1. Boxes represent the inter quartile ranges, lines inside the boxes denote medians, and circles are outliers. +, adjust *P* value <0.01; ns, not significant. Wilcoxon rank sum test. (PDF 547 kb)
Additional file 5: Figure S3. The gut microbial abundances of genera enriched in groups do not correlated with BMI, age or gender. The relative abundances of genera overrepresented (above 4) or deficient (below 11) in subjects for each control (*n* = 41), pHTN (*n* = 56), and HTN (*n* = 99) sample are shown. The information for BMI, age and gender of each participant are included in the heat map. (PDF 669 kb)
Additional file 6: Figure S4. The gut microbiome profile of HTN in the additional independent metagenomic analysis. (a) Bacterial *α* diversity (Shannon index) at the genus level is compared between control (*n* = 3) and HTN (*n* = 3) group. *P* value is from *t* test. (b) The genera abundance alteration in HTN patients is compared with the controls. Red represents more abundant, blue indicates less abundant. The genera marked with green points show a consistent trend in HTN compared with the results in stage 1 metagenomic analysis, while gray points represent the genera with inconsistent variation. (PDF 330 kb)
Additional file 7: Table S8. Detailed information for 1,120,526 gene markers. (XLSX 28022 kb)
Additional file 8: Figure S5. Size distribution and taxonomic assignment of CAGs. (a) The 1,120,526 genes significantly different across groups are clustered into linked gene groups, and the distribution of gene number within these clusters are shown in the histogram. Clusters with a gene number higher than 50 are defined as CAG. (b) Characterization of taxonomic assignment for CAGs based on the genes. The size of points denotes the gene number within the CAG, and the color of points indicates different phylum. The *X*-axis (coverage) represents the percentage of genes in the CAGs annotated to known bacterial phylum, and the *Y*-axis is the identity of genes to align with a genome in both DNA and protein sequences according to BLAST. (PDF 313 kb)
Additional file 9: Figure S6. The correlation between overrepresented CAGs and clinical indices of subjects including SBP, DBP, BMI, FBG, TC, TG and LDL. Spearman’s correlation analysis between CAGs and clinical factors is performed according to the relative abundance of CAGs and the data of clinical parameter. The color are scaled with the correlation coefficients, positive correlation is expressed in red, and negative correlation in blue.+, adjust *P* value <0.01; *, adjust *P* value <0.05. (PDF 826 kb)
Additional file 10: Figure S7. Random forest classification of pHTN, HTN and control using explanatory variables of CAGs + species. (a) ROC for the testing set consisted of controls, pHTN and HTN is performed based on the random forest model using the 1000 most important variables by ranking the variables by importance. The AUC is 0.67 for control versus pHTN (*n* = 12, red curve), AUC = 0.81 for control versus HTN (*n* = 12, green curve), and AUC is 0.47 for pHTN versus HTN (*n* = 13, blue curve). (b) The top 30 different CAGs or species distinguish HTN from control based on the random forest model. The bar lengths denote mean decrease accuracy, and the color represents CAGs or species enriched in control (blue), HTN (red), and neither (gray). (PDF 499 kb)
Additional file 11: Table S17. Characteristics of the donors for microbiota transplantation. The donors for microbiota transplantation consist of two patients of HTN and one normotensive control. (DOC 32 kb)
