# Supplementary material for: Gut microbiota dysbiosis contributes to the development of hypertension
Source: Microbiome. 2017 Feb 1;5:14. doi: 10.1186/s40168-016-0222-x (PMC5286796; doi:10.1186/s40168-016-0222-x)
Supplement: Additional file 1: Table S1. — Characteristics of the study cohort. A total of 196 participants consisted of 41 healthy controls, 56 subjects of pHTN, and 99 patients with HTN were enrolled. (DOC 42 kb) [file 40168_2016_222_MOESM1_ESM.doc]

| **Characteristics of the study cohort** | | | | | |
| --- | --- | --- | --- | --- | --- |
| characteristics | Control | pHTN | HTN | *P*-value  C vs P | *P*-value  C vs H |
| N | 41 | 56 | 99 |  |  |
| Gender (% female) | 21.95 | 7.14 | 6.06 |  |  |
| Age, y (S.D.) | 53.7 (5.9) | 51.8 (6.6) | 53.6 (5.5) | 0.24 | 0.88 |
| SBP, mmHg (S.D.) | 115.3 (7.4) | 127.9 (10.4) | 148.8 (14.2) | <0.001 | <0.001 |
| DBP, mmHg (S.D.) | 74.1 (6.5) | 82.4 (5.8) | 94.7 (9.2) | <0.001 | <0.001 |
| BMI, kg/m^2^ (S.D.) | 25.2 (3.3) | 25.3 (2.9) | 26 (3.5) | 0.85 | 0.15 |
| FBG, mmol/l (S.D.) | 5.3 (0.86) | 5.3 (0.67) | 5.8 (1.4) | 0.56 | 0.026 |
| TC, mmol/l (S.D.) | 5.4 (0.95) | 5.8 (3.2) | 5.6 (1.8) | 0.6 | 0.78 |
| TG, mmol/l (S.D.) | 1.7 (2.1) | 2.2 (2.6) | 2.1 (2.1) | 0.28 | 0.06 |
| HDL, mmol/l (S.D.) | 1.25 (0.25) | 1.3 (0.28) | 1.38 (0.4) | 0.47 | 0.1 |
| LDL, mmol/l (S.D.) | 2.7 (0.79) | 2.7 (0.7) | 3.8 (9.8) | 0.99 | 0.67 |

**Table S1.** Characteristics of the study cohort. A total of 196 participants consisted of 41 healthy controls, 56 subjects of pHTN, and 99 patients with HTN were enrolled. Control is defined as SBP ≤125 mmHg and DBP ≤80 mmHg for untreated subjects. pHTN is defined as 125 mmHg < SBP ≤139 mmHg or 80 mmHg < DBP ≤89 mmHg subjects without antihypertensive treatments. HTN is defined as SBP ≤140 mmHg or DBP ≤90 mmHg patients without antihypertensive treatments. N, sample size; C, control; P, pHTN; H, HTN; S.D., standard deviation; SBP, systolic blood pressure; DBP, diastolic blood pressure; BMI, body mass index; FBG, fasting blood glucose; TC, total cholesterol; TG, triglyceride; HDL, high density lipoprotein; LDL, low density lipoprotein. Other than SBP and DBP, there was no significant difference in other clinical parameters among groups, except for FBG.
