# Supplementary figures and images for: Gut microbiota dysbiosis contributes to the development of hypertension
Source: Microbiome. 2017 Feb 1;5:14. doi: 10.1186/s40168-016-0222-x (PMC5286796; doi:10.1186/s40168-016-0222-x)

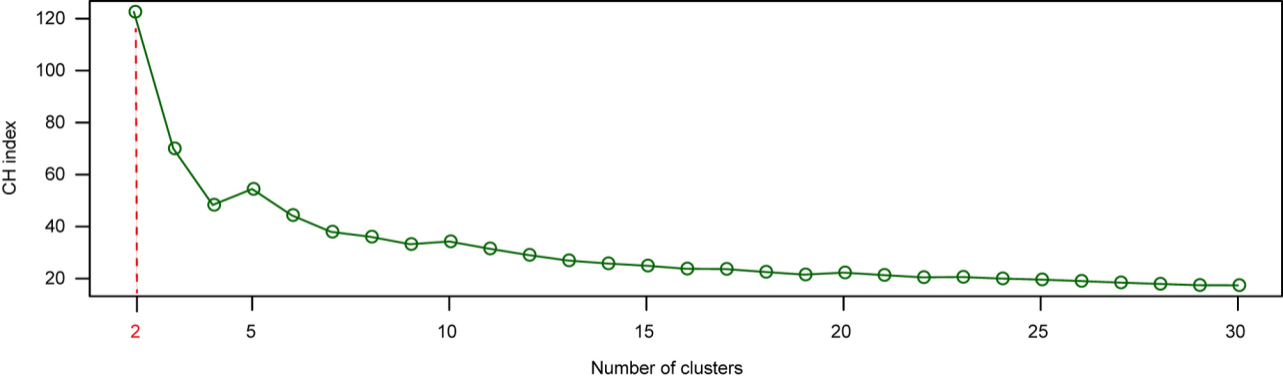

Supplement: Additional file 3: Figure S1. — The number of enterotypes in our cohort is most rational at 2. Based on the PAM clustering method, a total of 196 stool samples are clustered into different numbers of community types with CH index, which shows the performance in recovering cluster numbers. The maximum CH index at two clusters (enterotypes) indicates the optimal enterotype number. (PDF 183 kb) [file 40168_2016_222_MOESM3_ESM.pdf]

**a**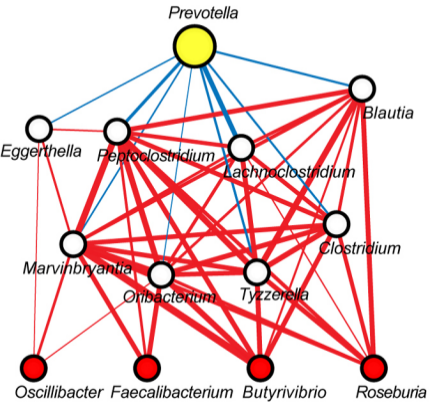**b**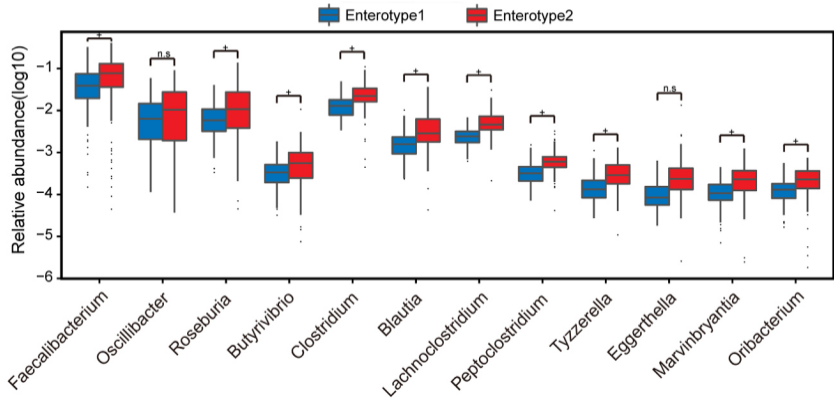

Supplement: Additional file 4: Figure S2. — The interaction network of genera in enterotype 1. (a) The main contributor in enterotype 1 is shown with yellow circle (Prevotella), genera shown by white circles link to it directly, and red ones indirectly. Edges between nodes in red denote Spearman’s correlation > 0.4, and correlation ≤0.4 is in blue. The width of edges is scaled by correlation index. (b) The twelve genera negatively correlated with Prevotella are all decreased in enterotype 1. Box plots are shown to compare the relative abundances of genera within the interaction network of enterotype 1. Ten out of twelve genera are significantly decreased in enterotype 1. Boxes represent the inter quartile ranges, lines inside the boxes denote medians, and circles are outliers. +, adjust P value <0.01; ns, not significant. Wilcoxon rank sum test. (PDF 547 kb) [file 40168_2016_222_MOESM4_ESM.pdf]

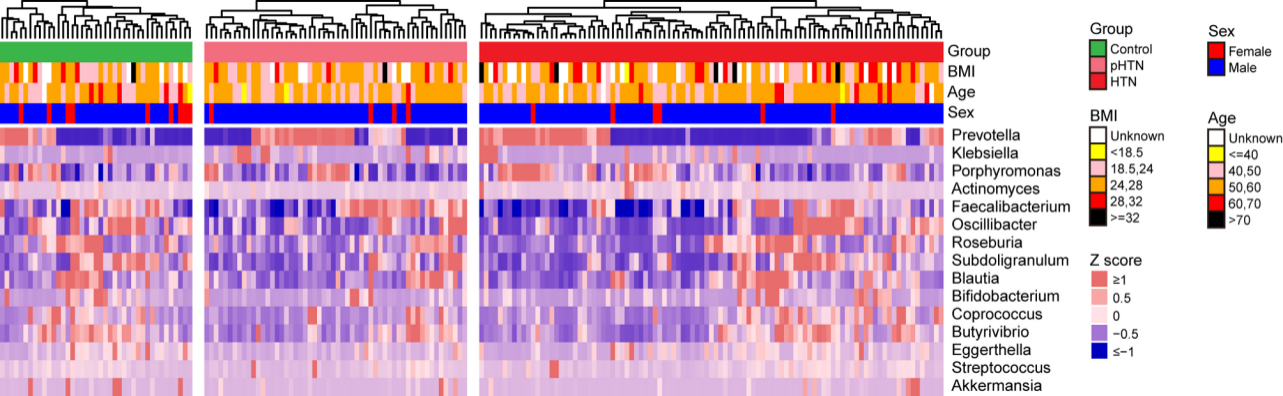

Supplement: Additional file 5: Figure S3. — The gut microbial abundances of genera enriched in groups do not correlated with BMI, age or gender. The relative abundances of genera overrepresented (above 4) or deficient (below 11) in subjects for each control (n = 41), pHTN (n = 56), and HTN (n = 99) sample are shown. The information for BMI, age and gender of each participant are included in the heat map. (PDF 669 kb) [file 40168_2016_222_MOESM5_ESM.pdf]

**a**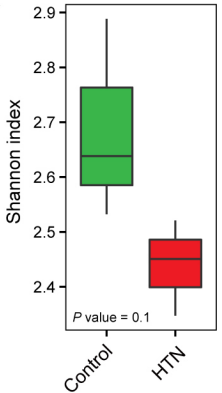**b**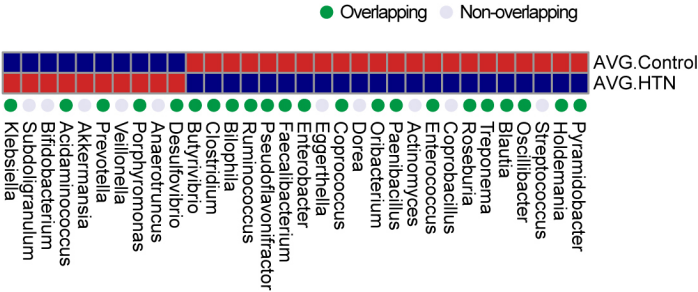

Supplement: Additional file 6: Figure S4. — The gut microbiome profile of HTN in the additional independent metagenomic analysis. (a) Bacterial α diversity (Shannon index) at the genus level is compared between control (n = 3) and HTN (n = 3) group. P value is from t test. (b) The genera abundance alteration in HTN patients is compared with the controls. Red represents more abundant, blue indicates less abundant. The genera marked with green points show a consistent trend in HTN compared with the results in stage 1 metagenomic analysis, while gray points represent the genera with inconsistent variation. (PDF 330 kb) [file 40168_2016_222_MOESM6_ESM.pdf]

**a**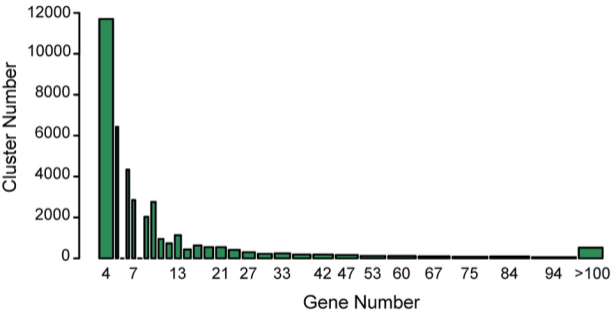**b**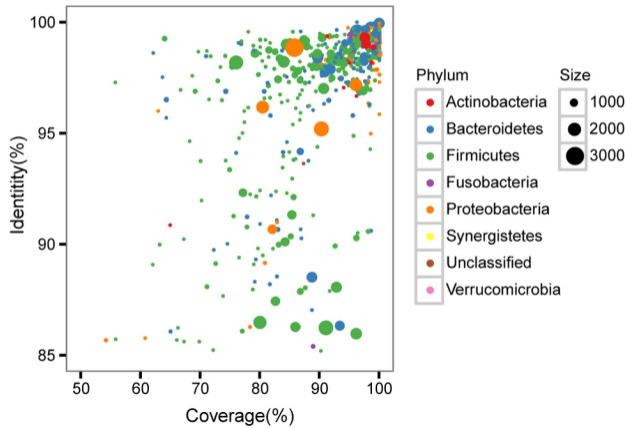

Supplement: Additional file 8: Figure S5. — Size distribution and taxonomic assignment of CAGs. (a) The 1,120,526 genes significantly different across groups are clustered into linked gene groups, and the distribution of gene number within these clusters are shown in the histogram. Clusters with a gene number higher than 50 are defined as CAG. (b) Characterization of taxonomic assignment for CAGs based on the genes. The size of points denotes the gene number within the CAG, and the color of points indicates different phylum. The X-axis (coverage) represents the percentage of genes in the CAGs annotated to known bacterial phylum, and the Y-axis is the identity of genes to align with a genome in both DNA and protein sequences according to BLAST. (PDF 313 kb) [file 40168_2016_222_MOESM8_ESM.pdf]

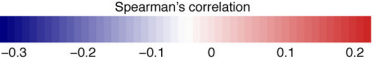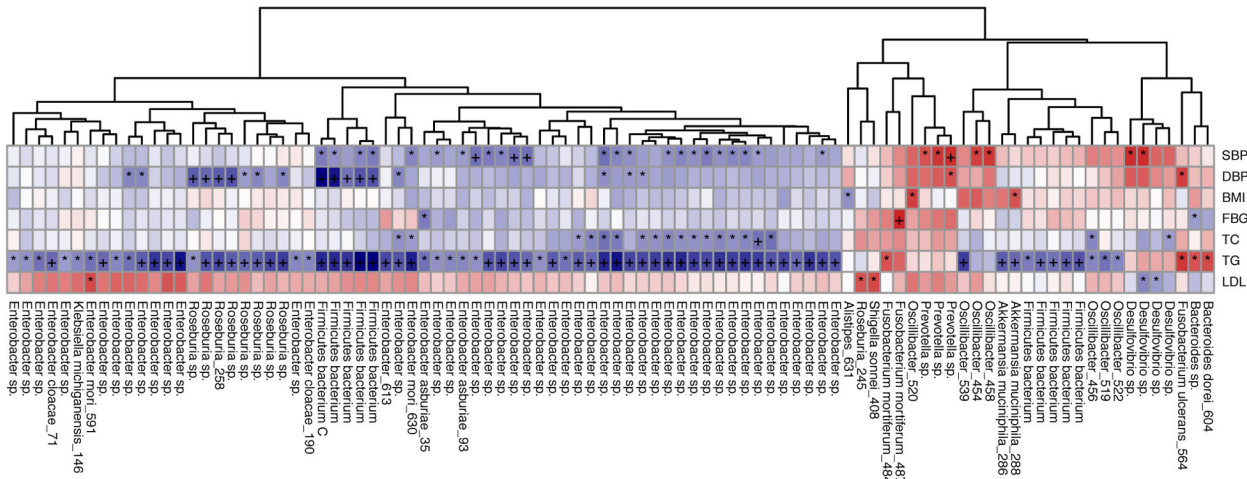

Supplement: Additional file 9: Figure S6. — The correlation between overrepresented CAGs and clinical indices of subjects including SBP, DBP, BMI, FBG, TC, TG and LDL. Spearman’s correlation analysis between CAGs and clinical factors is performed according to the relative abundance of CAGs and the data of clinical parameter. The color are scaled with the correlation coefficients, positive correlation is expressed in red, and negative correlation in blue.+, adjust P value <0.01; *, adjust P value <0.05. (PDF 826 kb) [file 40168_2016_222_MOESM9_ESM.pdf]

**a**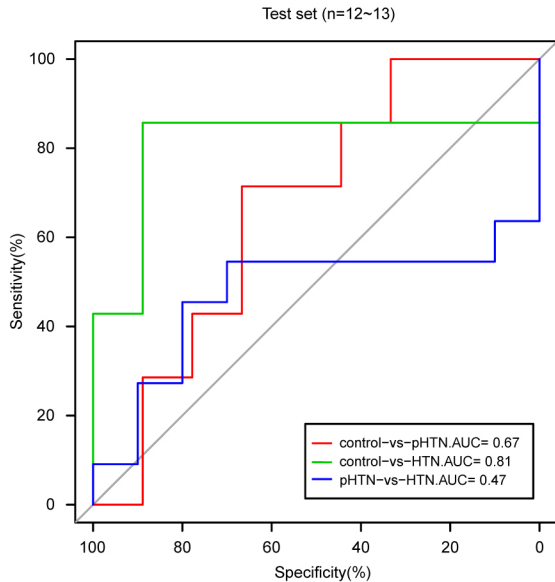**b**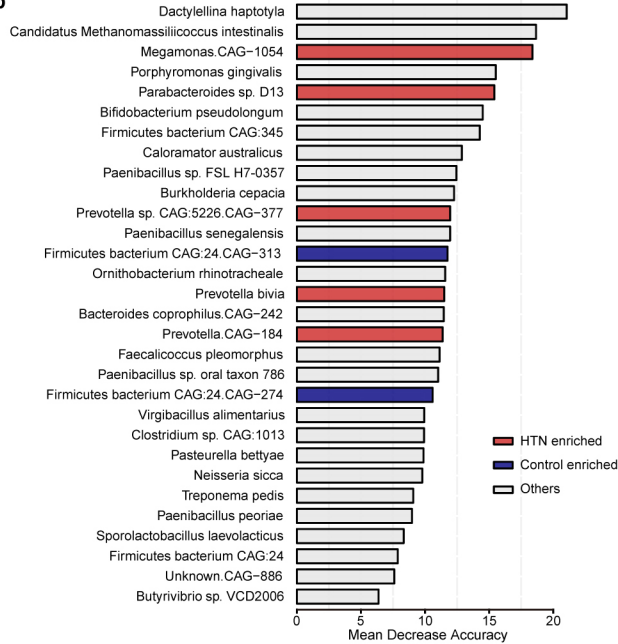

Supplement: Additional file 10: Figure S7. — Random forest classification of pHTN, HTN and control using explanatory variables of CAGs + species. (a) ROC for the testing set consisted of controls, pHTN and HTN is performed based on the random forest model using the 1000 most important variables by ranking the variables by importance. The AUC is 0.67 for control versus pHTN (n = 12, red curve), AUC = 0.81 for control versus HTN (n = 12, green curve), and AUC is 0.47 for pHTN versus HTN (n = 13, blue curve). (b) The top 30 different CAGs or species distinguish HTN from control based on the random forest model. The bar lengths denote mean decrease accuracy, and the color represents CAGs or species enriched in control (blue), HTN (red), and neither (gray). (PDF 499 kb) [file 40168_2016_222_MOESM10_ESM.pdf]
