# Supplementary material for: Gut microbiota dysbiosis contributes to the development of hypertension
Source: Microbiome. 2017 Feb 1;5:14. doi: 10.1186/s40168-016-0222-x (PMC5286796; doi:10.1186/s40168-016-0222-x)
Supplement: Additional file 11: Table S17. — Characteristics of the donors for microbiota transplantation. The donors for microbiota transplantation consist of two patients of HTN and one normotensive control. (DOC 32 kb) [file 40168_2016_222_MOESM11_ESM.doc]

| **Characteristics of the donors for microbiota transplantation** | | | |
| --- | --- | --- | --- |
| characteristics | Control | HTN1 | HTN2 |
| Gender | male | male | female |
| Age, y | 48 | 45 | 54 |
| SBP, mmHg | 119 | 157 | 160 |
| DBP, mmHg | 68 | 104 | 90 |
| BMI, kg/m^2^ | 23.94 | 25.2 | 22.89 |
| FBG, mmol/l | 5.35 | 5.78 | 5.3 |
| TC, mmol/l | 3.79 | 5.34 | 4.24 |
| TG, mmol/l | 0.62 | 1.41 | 0.57 |
| HDL, mmol/l | 0.89 | 1.06 | 0.93 |
| LDL, mmol/l | 2.21 | 3.25 | 2.65 |

**Table S17.** Characteristics of the donors for microbiota transplantation. The donors for microbiota transplantation consisted of two patients of HTN and one normotensive control. Control is defined as SBP ≤125 mmHg and DBP ≤80 mmHg for untreated subjects. Case of HTN is defined as SBP ≤140 mmHg or DBP ≤90 mmHg patients without antihypertensive treatments. SBP, systolic blood pressure; DBP, diastolic blood pressure; BMI, body mass index; FBG, fasting blood glucose; TC, total cholesterol; TG, triglyceride; HDL, high density lipoprotein; LDL, low density lipoprotein.
